# Hip dysplasia is not uncommon but frequently overlooked: a cross-sectional study based on radiographic examination of 1,870 adults

**DOI:** 10.1080/17453674.2021.1936918

**Published:** 2021-07-09

**Authors:** Rebecka Leide, Anna Bohman, Daniel Wenger, Søren Overgaard, Carl Johan Tiderius, Cecilia Rogmark

**Affiliations:** 1 Department of Clinical Sciences, Lund University, Lund, Sweden; 2 Department of Orthopedics, Halland Hospital, Halmstad, Sweden; 3 Department of Emergency Medicine, Central Hospital, Kristianstad, Sweden; 4 Department of Orthopedics, Skåne University Hospital, Lund and Malmö, Sweden; 5 Department of Clinical Medicine, University of Copenhagen, Copenhagen, Denmark; 6 Department of Orthopaedic Surgery and Traumatology, Copenhagen University Hospital, Bispebjerg, Denmark

## Abstract

Background and purpose — Hip dysplasia in adults is a deformity in which the acetabulum inadequately covers the femoral head. The prevalence is sparingly described in the literature. We investigated the prevalence in Malmö (Sweden) and assessed whether the condition was recognized in the radiology reports.

Subjects and methods — All pelvic radiographs performed in Malmö during 2007–2008 on subjects aged 20–70 years with a Swedish personal identity number were assessed. 1,870 digital radiographs were eligible for analysis. The lateral center-edge angle (LCEA) and acetabular index angle (AIA) were measured. Hip dysplasia was defined as an LCEA ≤ 20°. Intraclass correlation coefficients (ICC) for intra-observer measurements ranged from 0.87 (AIA, 95% CI 0.78–0.93) to 0.98 (LCEA, CI 0.97–0.99).

Results — The prevalence of hip dysplasia (LCEA ≤ 20°) was 5.2% (CI 4.3–6.3), (98/1,870). There was no statistically significant difference between the sexes for either prevalence of hip dysplasia or mean LCEA. The mean AIA was 0.9° (CI 0.3–1.3) higher in men (4.1 SD 5.5) compared with women (3.2 SD 5.4). The radiologists had reported hip dysplasia in 7 of the 98 cases.

Interpretation — The prevalence of hip dysplasia in Malmö (Sweden) is similar to previously reported data from Copenhagen (Denmark) and Bergen (Norway). Our results indicate that hip dysplasia is often overlooked by radiologists, which may influence patient treatment.

Note: Please check the heading levels

Hip dysplasia is an anatomical deformity defined by a reduced lateral center-edge angle (LCEA) expressing insufficient acetabular coverage of the femoral head. An angle ≤ 20° is considered pathologic, whereas an angle between 21° and 25° is said to be “borderline” (Wiberg 1939, Fredensborg 1976, Ogata et al. 1990, Jacobsen and Sonne-Holm 2005). The acetabular index angle (AIA) describes the slope of the acetabular roof (Tönnis 1976) and a normal range has been suggested as 3° to 13° (Tannast et al. 2015a). Adult hip dysplasia ranges from being an asymptomatic anatomic variation to a painful disease. Diagnosis requires referral for an anteroposterior (AP) radiograph of the pelvis. Although the radiographic measurements have been known for decades, a diagnostic delay is common as radiologists and clinicians often overlook the deformity (Nunley et al. 2011).

The prevalence of hip dysplasia varies from 2% to 8% in the few previous studies and the definition of the diagnosis based on the LCEA is inconsistent (Croft et al. 1991, Smith et al. 1995, Inoue et al. 2000, Jacobsen and Sonne-Holm 2005, Engesaeter et al. 2013). The prevalence has not been studied in Sweden before. In an international comparison, we perceive adult hip dysplasia to be a seldom discussed diagnosis in Sweden. Therefore, we determined the prevalence of hip dysplasia in Malmö, an urban area in southern Sweden, and investigated whether hip dysplasia was recognized in radiologists’ reports.

## Subjects and methods

### Study design and population

For this retrospective cross-sectional study, all AP pelvic radiographs performed during 2007–2008 at Skåne University Hospital in Malmö were assessed for eligibility (n = 10,658). Inclusion criteria were a Swedish personal identity number and age 20–70 years. The age span was chosen to ensure full skeletal maturity and to diminish the risk of age-associated degenerative changes that could influence the measurement quality. The study period (2007–2008) and the requirement of a Swedish personal identity number were chosen to enable future long-term follow-up of cases identified with hip dysplasia. We included only the 1st image in subjects with repeated radiographic examinations during the study period.

The following exclusion criteria were applied during radiographic assessment of the remaining radiographs: foramen obturator index outside 0.7–1.8 (see section “Radiographic measurements”), osteoarthritis (OA), hip implants, hip fracture, acetabular fracture, major skeletal tumor, grossly displaced pelvic fracture, a history of childhood hip disorder, inflammatory joint disease, avascular necrosis of the femoral head, skeletal deformity in the hip joint due to neurological disorder, and poor imaging quality. OA was considered present if there was joint space narrowing on the right and/or left side. The assessment was based on information available in the referral, the radiology report, and the observer’s (RL: MD, resident orthopedic surgeon and PhD student in orthopedics) own assessment of the radiographic image. In uncertain cases, images were discussed with a senior orthopedic consultant (CJT, SO, or CR).

Subjects’ age, sex, the reason for referral and the radiologist’s statements were collected. Referral reports were read to divide the material into trauma (n = 928) and non-trauma (n = 2,113). The referral reports and radiology statements were digital and available in immediate connection with the radiographs, i.e., no reports were lost.

**Figure 1.**
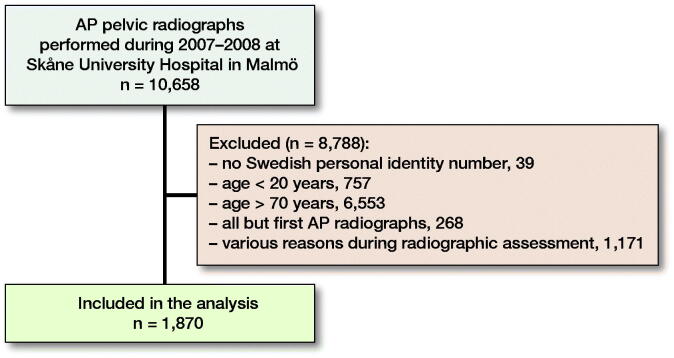
Flowchart of exclusion steps resulting in the study population of 1,870 subjects. For detailed exclusion criteria see “Study design and population”.

During assessment, 117 subjects were found to only have a referral for radiographic examination. As these subjects never underwent radiographic examination, the issue was considered as an error in the archive rather than missing data. After finalizing the exclusion steps, 1,870 subjects were eligible for inclusion (Figure 1).

### Radiographic measurements

The normal routine at Skåne University Hospital in Malmö is to include an AP projection of the pelvis when performing radiographic examination of the hip. An AP of the pelvis is necessary to enable measurement of the LCEA.

**Figure 2.**
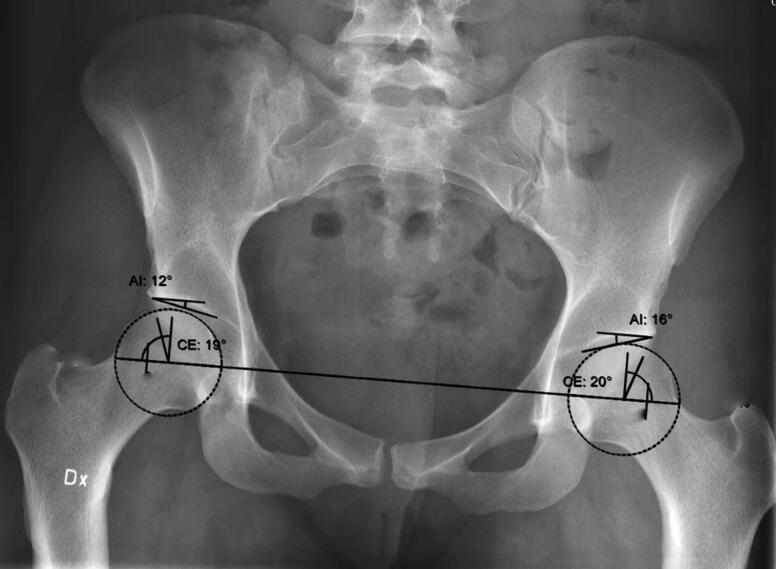
Example of output using the 2D dysplasia guide of the Sectra Planning System. AI = acetabular index angle (termed AIA in the text); CE = centre edge angle (termed LCEA in the text).

The LCEA was defined as the angle between 2 lines drawn through the center of the femoral head, the 1st line perpendicular to the horizontal line and the 2nd line drawn to the lateral subchondral sclerotic zone of the acetabular roof, the so-called “sourcil.” The horizontal line was defined as the line between the center of the femoral heads, according to Wiberg’s original description of the LCEA (Wiberg 1939) (Figure 2).

The AIA was defined as the angle between the horizontal line and a line between the lateral and medial margin of the sourcil (Tönnis 1976), and was used as a complementing description of the anatomy.

To determine the degree of pelvic rotation in the axial plane, the foramen obturator index (FOI) was used. FOI equals the widest horizontal diameter of the right foramen obturator divided by the widest horizontal diameter of the left foramen obturator (Tönnis 1976). A FOI between 0.7 and 1.8 is recommended when assessing the LCEA, as greater pelvic rotation may affect LCEA measurements (Jacobsen et al. 2004).

Measurements were performed by a single observer (RL) using a hip dysplasia guide in the radiography software (see below), where the LCEA and AIA are obtained in whole degrees. 

### Prevalence of hip dysplasia

Prevalence was defined as the proportion of the study population with hip dysplasia in 1 or both hips. Hip dysplasia was defined as an LCEA ≤ 20° and borderline hip dysplasia was defined as an LCEA of ≤ 25°.

### Mention of hip dysplasia in radiology reports

Radiology reports were read to register whether the presence of hip dysplasia was mentioned, either in exact terms or by describing typical features.

### Intra-observer reliability

To assess the intra-observer reliability of the LCEA and AIA measurements, repeated measures on 50 randomly selected subjects were performed by RL 2 months after finalizing the 1st assessment. The intraclass correlation coefficients (ICC) for the LCEA were excellent (> 0.9) according to Koo and Li (2016); 0.98 (CI 0.97–0.99) for both right and left hips. The ICC for the AIA was good (0.75–0.9) according to Koo and Li (2016) for both hips; 0.87 (CI 0.79–0.93) for right hips and 0.87 (CI 0.78–0.93) for left hips (Table 1). No 2nd reading was performed to assess inter-observer reliability.

**Table 1.** Intra-observer reliability for the LCEA and AIA

	Systematic	Random	
	error (°)	error (°)	ICC (95% CI)
LCEA right hip	0.05	0.62	0.98 (0.97–0.99)
LCEA left hip	0.06	0.57	0.98 (0.97–0.99)
AIA right hip	–0.33	1.56	0.87 (0.79–0.93)
AIA left hip	–0.35	1.57	0.87 (0.78–0.93)

LCEA = lateral center-edge angle; AIA = acetabular index angle;

ICC = intraclass correlation coefficient, CI = confidence interval.

### Statistics and software

Before data collection, a power analysis showed that 1,400 subjects were needed to obtain a dysplasia prevalence with a precision of ±1% unit.

Means (SD) are presented for normally distributed variables, and medians (range) are presented for non-normally distributed variables. 95% confidence intervals (CI) were calculated. Continuous variables were considered normally distributed based on visual appearance of histograms, similarity between median and mean value, and skewness between –3 and 3. The normal distributions of the LCEA and AIA are presented together with a range of minus and plus 2 SDs from the mean. For inferential statistics, a significance level of < 0.05 was chosen.

The Mann–Whitney U-test was used for group comparison of non-normally distributed variables between independent groups. For group comparison of normally distributed variables, Student’s t-test for independent samples (2-tailed) was used for independent groups and paired t-test (2-tailed) for dependent groups. Pearson’s correlation coefficient was calculated to estimate correlation between the LCEA and AIA. A chi-square test was used to compare prevalences between groups. The Clopper–Pearson method was used to calculate 95% CI for proportions. To describe the intra-observer reliability for LCEA and AIA measurements, the systematic error = (mean of measurement 1 – mean of measurement 2)/2, random error = SD((measurement 1 – measurement 2)/√2), and ICC were calculated. ICC estimates were calculated with 95% CI and interpreted according to Koo and Li (2016).

Statistical analyses were performed with IBM SPSS Statistics version 25 (IBM Corp, Armonk, NY, USA). Radiographs were stored and viewed using Sectra PACS (Sectra IDS7 v21.1 Sectra AB, Linköping, Sweden). Radiographic measurements were performed using the dysplasia guide of the Sectra 2D Planning System (Sectra Orthostation Package, version 10.1).

### Ethics, funding, and potential conflicts of interest

The study was approved by the Regional Ethics Review Board 2016-01-26 (2015/910). Subjects’ consent was of opt-out type. Funding was received from the Greta and Johan Kock Foundation, Erik and Angelica Sparre Foundation, Swedish Research Council funding for clinical research in medicine (ALF), and Skåne University Hospital Foundation. None of the authors have any conflicts of interest to declare.

### Report

The STROBE guidelines for cross-sectional studies were used for the reporting of this study.

## Results

### Demographics

The median age of the 1,870 included subjects was 53 years (20–70) and 63% (n = 1,171) were female. The 1,171 subjects who were excluded after radiographic assessment were older, 58 years (20–70), p < 0.001. 28% (n = 530) of the included subjects were examined due to trauma and the rest for other reasons.

### Prevalence of hip dysplasia and mention of hip dysplasia in radiology reports

We found 98 subjects with hip dysplasia, resulting in a prevalence of 5.2%. 23% (n = 23) of these had bilateral findings (Table 2). There was no statistically significant difference in prevalence between women and men, 5.6% vs. 4.6% (Table 2). Nor was there any statistically significant difference in prevalence between subjects who were examined due to trauma compared with subjects who were examined for other causes, 6.4% (CI 4.5–8.8) vs. 4.8% (CI 3.7–6.1). 21% (n = 400) had borderline hip dysplasia (Table 2). In 91 of the 98 cases with hip dysplasia, there was no comment on the condition in the radiology report.

**Table 2.** Prevalence of hip dysplasia (LCEA ≤ 20°), and borderline hip dysplasia (LCEA ≤ 25°) in 1,870 subjects (699 men/1,171 women). Values are count, prevalence (%) with 95% confidence interval

	Either		Bilateral		Right		Left	
LCEA ≤ 20°								
Total	98	5.2 (4.3–6.3)	23	1.2 (0.8–1.8)	82	4.4 (3.5–5.4)	39	2.1 (1.5–2.8)
Men	32	4.6 (3.2–6.4)	5	0.7 (0.2–1.7)	25	3.6 (2.3–5.2)	12	1.7 (0.9–3.0)
Women	66	5.6 (4.4–7.1)	18	1.5 (0.9–2.4)	57	4.9 (3.7–6.3)	27	2.3 (1.5–3.3)
LCEA ≤ 25°								
Total	400	21 (20–23)	150	8.0 (6.8–9.3)	331	18 (16–20)	219	12 (10–13)
Men	147	21 (18–24)	48	6.9 (5.1–9.0)	120	17 (14–20)	75	11 (8.5–13)
Women	253	22 (19–24)	102	8.7 (7.2–11)	211	18 (16–20)	144	12 (11–14)

LCEA = lateral center-edge angle

### Radiographic measurements

**Figure 3.**
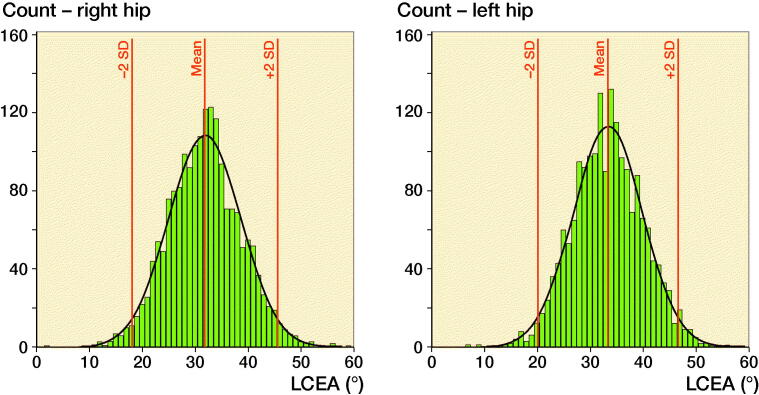
Distribution of the right and left LCEA in 1,870 adults. For right hips, the mean LCEA was 32 (SD 6.9) and the range of 2 SDs 18.1–45.6. For left hips, the mean LCEA was 33 (SD 6.6) and the range of 2 SDs 20.2–46.7.

There was no statistically significant sex-related difference regarding the mean LCEA; female mean LCEA 33° (SD 6.6), male mean LCEA 32° (SD 5.8), mean difference 0.5 (CI -0.1–1.0). Right hips had 1.6° (CI 1.4–1.8) lower LCEA than left hips, 32° (SD 6.9) vs. 33° (SD 6.6) (Figure 3).

**Figure 4.**
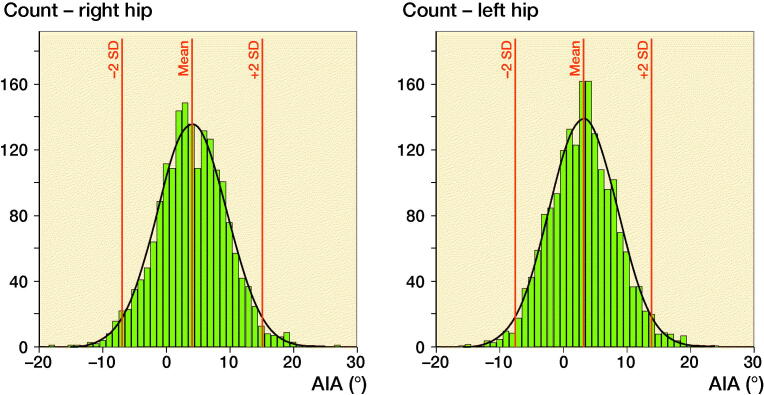
Distribution of the right and left AIA in 1,870 adults. For right hips, the mean AIA was 4.1 (SD 5.5) and the range of 2 SDs –6.9 to 15.0. For left hips, the mean AIA was 3.2 (SD 5.4) and the range of 2 SDs –7.7 to 13.9.

The mean AIA was 4.2° (SD 4.7) in male subjects and 3.3° (SD 5.1) in female subjects; mean difference was 0.9° (CI 0.4–1.3). Right hips had 0.9° (CI 0.7–1.1) higher AIA compared with left hips; 4.1° (SD 5.5) vs. 3.2° (SD 5.4) (Figures 3 and 4). There was a strong, negative correlation between the LCEA and AIA; Pearson’s correlation coefficient was –0.77 (p < 0.001) for right hips and –0.76 (p < 0.001) for left hips. Among hips with hip dysplasia (LCEA ≤ 20°), the mean AIA was 13.3° (SD 4.3) for right hips (n = 82), and 13.9° (SD 3.8) for left hips (n = 39), respectively.

## Discussion

A long tradition of clinical screening for developmental dysplasia of the hip (DDH) in neonates (Wenger et al. 2019) may have led to the misconception that adult hip dysplasia is not a concern in Sweden. Adult hip dysplasia and DDH have pathoanatomic similarities, but the link between them, if there is one, is not clearly understood. We found a prevalence of 5.2% for adult hip dysplasia in our cohort and only 7% of the identified cases were described in the radiology reports.

Our results were based on an LCEA ≤ 20°, which is the same definition as in a Danish study that reported results in line with ours; 5.4% (Jacobsen and Sonne-Holm 2005). A Norwegian study, which used a cut-off of a LCEA < 20°, reported a prevalence of 3.3% among 19-year-olds (Engesaeter et al. 2013). Comparisons with other studies are more difficult because their radiographic measurements were assessed on urograms and in most cases also with higher cut-off values: Croft et al. (1991) reported a prevalence of a LCEA ≤ 20° of 1.6% in a male population, Smith et al. (1995) a prevalence of an LCEA < 25° of 3.8% in a female population and Inoue et al. (2000) a prevalence of an LCEA < 25° of 8.1% among Japanese subjects and 2.9% among French subjects. Regarding the AIA, we found a clear negative correlation with the LCEA, which is in accordance with previous studies (Werner et al. 2012, Zhang et al. 2020).

The cut-off values 20° and 25° were originally proposed by Wiberg (1939) in relation to hip development following childhood hip disease. If borderline dysplasia (LCEA ≤ 25°) is to be considered pathologic, the prevalence in both our cohort and the Norwegian cohort (Engesaeter et al. 2013) would have been around 20%.

Inoue et al. (2000) reported women as having a higher prevalence than men in French and Japanese adults, using a cut-off value of LCEA < 25°. Engesaeter et al. (2013) concluded the same in Norwegian 19-year-olds, suggesting the prevalence for women to be 4.3% and for men 2.4%, using the cut-off value LCEA < 20°. We did not find any statistically significant difference between men and women in our study. This may be explained by different inclusion criteria, real differences between populations, and/or a type II error. In the case of a falsely accepted null hypothesis, a prevalence that differs only a few percentage units between sexes would not be clinically significant.

As suggested before (Engesaeter et al. 2013), we found right hips more often to be dysplastic than left hips. Along with that, we found a higher AIA in right hips, indicating a steeper acetabulum. In addition, re-directional periacetabular osteotomy (PAO) was more frequently performed on right hips compared with left hips (770 vs. 615) in a recent prospective study of PAO outcome (Larsen et al. 2020). Together, these results indicate that adult hip dysplasia may be more common in right hips.

We investigated whether or not the hip dysplasia was detected during radiographic examination in standard care. To our knowledge, this has not been previously studied. Our results suggest that a vast majority of hip dysplasia cases may be overlooked by Swedish radiologists. Underreading error has been shown to be among the most common of radiological errors, and delayed diagnosis due to radiological errors is most frequent in the musculoskeletal section (Kim and Mansfield 2014). In the current aspect, underreading of hip dysplasia may be reduced by education and raised awareness of the diagnosis among radiologists. Another important factor is for the referring clinician to ask for signs of dysplasia in relevant cases, i.e., awareness of the diagnosis needs to be increased amongst general practitioners and orthopedic surgeons as well.

We acknowledge some limitations to this study. We did not adjust for pelvic tilt (Wiberg 1939, Jacobsen et al. 2004). The distance between the coccyx and the symphysis can be used for this purpose, but has been considered difficult to identify on radiographs (Laborie et al. 2011) and does not affect the LCEA to a clinically relevant extent (Tannast et al. 2015b). OA was not scored according to a common classification system such as Kellgren and Lawrence (K&L). It is therefore possible that some individuals with a K&L grade 1 were included in the study. However, we have no reason to assume that this has influenced our results. Moreover, as hip dysplasia is a risk factor for OA (Jacobsen and Sonne-Holm 2005), exclusion of subjects with OA might lower the prevalence of hip dysplasia. However, it was a deliberate choice of exclusion as measurement of the LCEA has been shown to be affected by degenerative changes (Ipach et al. 2014). Furthermore, the radiographic assessments and measurements were performed by a resident orthopedic surgeon and not a radiologist. Studies have shown good inter-observer reliability for radiographic measurements between observers with varying experience (Tiderius et al. 2004, Herngren et al. 2018) and that radiologic technologists with appropriate training can interpret radiographs accurately (Piper et al. 2005, Woznitza et al. 2018). In our study, the observer had extensive training prior to the data collection and continuous support from senior orthopedic consultants during the readings. Lastly, the cohort consists of subjects who actively sought medical care, and not a random sample of the population. We believe our finding of similar prevalence amongst trauma and non-trauma radiographs speaks in favor of our sample being close to the general population and thus has not biased our results. By assessing the referrals, a number of the non-trauma cases were found to have back pain, knee pain, and screening for bone metastases as cause for their pelvic radiographic examination. If the group with non-trauma radiographs had only consisted of individuals with apparent hip symptoms, we might have found a higher prevalence of hip dysplasia compared with the group with trauma-related radiographs.

## Conclusion

Hip dysplasia was present in 5.2% of our cohort consisting of Swedish adults. The condition was mentioned in the radiology report in less than 1 in 10 cases with hip dysplasia, indicating that neither the referring clinicians nor the patients were informed of this potential cause of symptoms from the hip area.

In perspective, our findings indicate a need for raised awareness of how common adult hip dysplasia is, so that general practitioners, orthopedic surgeons, and radiologists take it into account as a differential diagnosis during examination, referral, and radiographic evaluation. Among Swedish patients with adult hip dysplasia, there is probably an unmet need for proper information concerning their condition, including treatment alternatives for those with symptoms.
